# Supplementary material for: NT3/TrkC Pathway Modulates the Expression of UCP-1 and Adipocyte Size in Human and Rodent Adipose Tissue
Source: Front Endocrinol (Lausanne). 2021 Mar 18;12:630097. doi: 10.3389/fendo.2021.630097 (PMC8015941; doi:10.3389/fendo.2021.630097)
Supplement: Supplementary file 1 [file DataSheet_1.docx]

**Supplemental material**

**Table S1.** Demographical characteristics and biochemical profile of the subjects included in the study grouped by the way of life

|  | **Active (n = 18)** | **Sedentary (n = 10)** | ***P*** |
| --- | --- | --- | --- |
| **Age, years** | 73.1± 2.9 | 74.5 ± 3.0 | 0.75081 |
| **BMI,** kg/m^2^ | 25.3 ± 1.0 | 25.9 ± 0.9 | 0.67988 |
| **Glucose,** mg/dL | 92.6 ± 3.9 | 104.9 ± 7.5 | 0.11677 |
| **Total cholesterol,** mg/dL | 184.6 ± 6.7 | 178.4 ± 14.1 | 0.65633 |
| **HDL-cholesterol,** mg/dL | 53.5 ± 3.6 | 54.4 ± 36.6 | 0.89070 |
| **LDL-cholesterol,** mg/dL | 103.8 ± 5.5 | 90.8 ± 10.8 | 0.24762 |
| **Triglycerides,** mg/dL | 122.9 ± 15.0 | 98.8 ± 21.4 | 0.35524 |

*P values obtained by Student’s t test*

**Table S2.** Demographical characteristics and biochemical profile of the subjects included in the study grouped by the presence or not of diabetes

|  | **Non diabetic (n = 21)** | **Diabetic (n = 7)** | ***P*** |
| --- | --- | --- | --- |
| **Age, years** | 72.9± 2.4 | 75.7 ± 4.3 | 0.56942 |
| **BMI,** kg/m^2^ | 25.8 ± 0.8 | 24.7 ± 1.6 | 0.52029 |
| **Glucose,** mg/dL | 96.1 ± 3.1 | 99.6 ± 12.4 | 0.69902 |
| **Total cholesterol,** mg/dL | 184.7 ± 8.0 | 175.4 ± 10.9 | 0.54707 |
| **HDL-cholesterol,** mg/dL | 55.6 ± 4.1 | 48.8 ± 3.5 | 0.34822 |
| **LDL-cholesterol,** mg/dL | 95.0 ± 5.9 | 112.0 ± 9.1 | 0.14427 |
| **Triglycerides,** mg/dL | 124.4 ± 15.7 | 84.0 ± 5.2 | 0.15718 |

*P values obtained by Student’s t test*

**Table S3.** Demographical characteristics and biochemical profile of the subjects included in the study grouped by the presence or not of hypertension

|  | **Normotensive (n = 13)** | **Hypertensive (n = 15)** | ***P*** |
| --- | --- | --- | --- |
| **Age, years** | 77.5± 1.9 | 70.2 ± 3.5 | 0.08766 |
| **BMI,** kg/m^2^ | 26.3 ± 1.1 | 24.8 ± 1.0 | 0.32745 |
| **Glucose,** mg/dL | 97.3 ± 5.1 | 96.7 ± 5.6 | 0.94061 |
| **Total cholesterol,** mg/dL | 183.0 ± 9.9 | 181.9 ± 8.9 | 0.93277 |
| **HDL-cholesterol,** mg/dL | 49.4 ± 2.8 | 58.2 ± 5.5 | 0.16588 |
| **LDL-cholesterol,** mg/dL | 98.2 ± 8.3 | 101.1 ± 6.5 | 0.78466 |
| **Triglycerides,** mg/dL | 146.4 ± 22.8 | 84.5 ± 6.0 | **0.01195*** |

*P values obtained by Student’s t test*
